# The chemokines CXCL12 and CXCL14 differentially regulate connective tissue markers during limb development

**DOI:** 10.1038/s41598-017-17490-z

**Published:** 2017-12-08

**Authors:** Sonya Nassari, Cédrine Blavet, Marie-Ange Bonnin, Sigmar Stricker, Delphine Duprez, Claire Fournier-Thibault

**Affiliations:** 1 Sorbonne Universités, UPMC Univ Paris 06, CNRS UMR7622, Inserm U1156, IBPS-Developmental Biology Laboratory, F-75005 Paris, France; 2 Institute for Chemistry and Biochemistry, Freie Universität Berlin, Berlin, Germany

## Abstract

Connective tissues (CT) support and connect organs together. Understanding the formation of CT is important, as CT deregulation leads to fibrosis. The identification of CT specific markers has contributed to a better understanding of CT function during development. In developing limbs, *Osr1* transcription factor is involved in the differentiation of irregular CT while the transcription factor *Scx* labels tendon. In this study, we show that the CXCL12 and CXCL14 chemokines display distinct expression pattern in limb CT during chick development. CXCL12 positively regulates the expression of *OSR1* and *COL3A1*, a collagen subtype of irregular CT, while CXCL14 activates the expression of the tendon marker *SCX*. We provide evidence that the CXCL12 effect on irregular CT involves CXCR4 receptor and vessels. In addition, the expression of *CXCL12, CXCL14* and *OSR* genes is suppressed by the anti-fibrotic BMP signal. Finally, mechanical forces, known to be involved in adult fibrosis, control the expression of chemokines, CT-associated transcription factors and collagens during limb development. Such unexpected roles of CXCL12 and CXCL14 chemokines during CT differentiation can contribute to a better understanding of the fibrosis mechanisms in adult pathological conditions.

## Introduction

In the body, the main role of connective tissues (CT) is to support organs and to connect cells and tissues. CT are primarily composed of extracellular matrix produced by fibroblasts that derive from mesenchymal progenitor cells during development. Several types of CT exist: specialized CT refers to bone and cartilage, while dense CT is divided into regular CT, which corresponds to tendon and ligament and irregular CT (ICT). ICT includes CT surrounding organs, as for example the perichondrium around cartilage and the epimysium around muscles, and CT inside organs, as for example muscle connective tissue (MCT)^1^. Understanding the specification and differentiation processes of CT types from undifferentiated mesenchymal cells is important, as CT deregulation leads to fibrosis, a process attributed to excess deposition of extracellular matrix in response to injury, inflammation or aging^2^. Fibrosis is also a major pathological feature of chronic autoimmune diseases, tumour invasion and progressive myopathies^2^.

Until a few years ago, studying the specification of CT types during embryogenesis was still challenging, due to the absence of specific markers to distinguish the different forms of CT. Most studies designed to identify CT-specific genes during development have been conducted on the vertebrate limb musculoskeletal system as a model. Indeed, this multicomponent structure formed by skeletal muscle, bone and cartilage (specialized CT), tendon and ligament (regular CT) and irregular CT (ICT), surrounding and connecting the different elements of the musculoskeletal system, is particularly suitable to investigate CT differentiation in the embryo. In limbs, all types of CT derive from the lateral plate mesoderm^3,4^, while myogenic cells originate from the somitic dermomyotome and migrate into the limb buds^3,5^. Classical embryological approaches have shown that limb lateral plate mesoderm contains positional information cues for limb formation^6,7^. The limb mesenchyme also influences muscle patterning^8^, highlighting the importance of CT derivatives for limb musculoskeletal morphogenesis. In the developing limb, studies on CT differentiation first focused on collagen, the major component of the extracellular matrix and showed that type I and type III collagens are both expressed in dense regular and irregular CT^9^. However, type I collagen becomes progressively predominant in tendons, while both type III and type VI collagens mostly characterize both ICT and MCT^9–11^.

More recently, the identification of specific markers and genetic tools allowing the labelling and manipulation of CT fibroblasts has largely contributed to a better understanding of the role of CT types during limb development. The *Scleraxis* (*Scx*) gene, encoding a bHLH transcription factor, is expressed in tendon and ligament cells^12^ and *Scx*−/− mice showed severe defects in force-transmitting tendons^13^. *Scx* consequently characterizes dense regular CT (tendon and ligament). In addition, *Scx* has been shown to activate *Col1a1* transcription, coding for one chain of the main collagen expressed in tendon cells^14^. In contrast to tendon and ligament, the characterization of ICT and MCT remains challenging. *Tcf4*, a member of the *Tcf/Lef* family of transcription factors, is highly expressed in MCT during chick and mouse limb development^15,16^. *TCF4* gain- and loss-of-function experiments demonstrated that *TCF4*-expressing cells contribute to limb muscle patterning in chick embryos^15^. Similarly, mispatterning of limb muscles and tendons are observed after ablation of the T-box transcription factor *Tbx5* in mouse lateral plate-derived cells^17^. Interestingly, *Scx* and *Tcf4* genes have been shown to be critical regulators of fibrosis in different adult organs^18,19^. However, although *Tcf4* and *Tbx5* are considered as MCT-associated markers, *Tcf4* is also expressed in myogenic cells^16^ and *Tbx5* is also observed in cartilage, tendon and muscle progenitors of mouse limbs^20^, showing that both factors are not specific to ICT and MCT. More recently, the zinc finger transcription factors Odd Skipped-related-1 and -2 (*Osr1* and *Osr2)* have been identified as being expressed in ICT during limb development, with a prevalence of *Osr2* expression in MCT^21^. *Osr1* and *Osr2* genes are not expressed in tendon, ligament and cartilage, with the exception of the presumptive joints^21^. Both *Osr1* and *Osr2* drive ICT differentiation at the expense of cartilage and tendon and are required for the differentiation of ICT fibroblasts^22^. Overexpression of *OSR1* or *OSR2* in chick limb mesenchymal cells induces the expression of ICT markers such as *COL3A1* and *COL6A1*, while downregulating the expression of cartilage and tendon markers. Conversely, *OSR1* or *OSR2* inactivation downregulates *COL3A1* and *COL6A1* expression, while increasing cartilage formation in chick limb cells^22^.

During limb development, the expression of these CT-associated transcription factors is regulated by extrinsic signalling pathways. BMP4 represses *Tcf4* ^23^ and *Scx* expression^12^, while *SCX* expression is positively regulated by FGF4^24,25^. Importantly, both BMP and FGF signalling pathways are involved in the regulation of adult tissue fibrosis^26,27^. Chemokines are also important regulators of fibrosis^28^ and interestingly, CXCL12 and CXCL14 have been shown to delineate some CT subpopulations in embryonic limbs^29–31^. Identified CXCL12 functions during embryogenesis include essential roles in the migration process of hematopoietic stem cells^32,33^, neurons^34,35^, germ cells^36,37^ and skeletal muscle cells^29^. These functions are mediated by two chemokine receptors, CXCR4 and CXCR7 that signal individually in different cells or act in a cooperative manner in the same cell^38^. The role of CXCL14 during development remains more elusive but CXCL14 possesses chemoattractive activity for activated macrophages, immature dendritic cells and natural killer cells^39^ and it has been shown that CXCL14-forced expression suppresses tumour growth in mice^40,41^. Specific receptors for CXCL14 have not yet been identified but it is proposed that CXCL14 is a natural inhibitor of the CXCL12/CXCR4 axis^42^.

In this study, we show that CXCL12 and CXCL14 chemokines display distinct expression pattern in limb CT and regulate the expression of specific CT markers. We provide evidence that CXCL12 positively regulates the expression of *OSR1* and *COL3A1*, a major collagen subtype of the ICT, in chick embryonic limbs and fibroblasts, while CXCL14 activates the expression of the tendon marker *SCX* in chick fibroblasts. Moreover, the expression of *CXCL12, CXCL14* and *OSR* genes is negatively regulated by BPM4, while the blockade of BMP activity is sufficient for enhancing *CXCL12, CXCL14* and *OSR1* expression. Lastly, the expression of *CXCL12*, *CXCL14* and CT markers is decreased in the absence of muscle contraction.

## Results

### *CXCL12* and *CXCL14* chemokines display distinct expression pattern in limb CT during chick development

**Figure 1:**
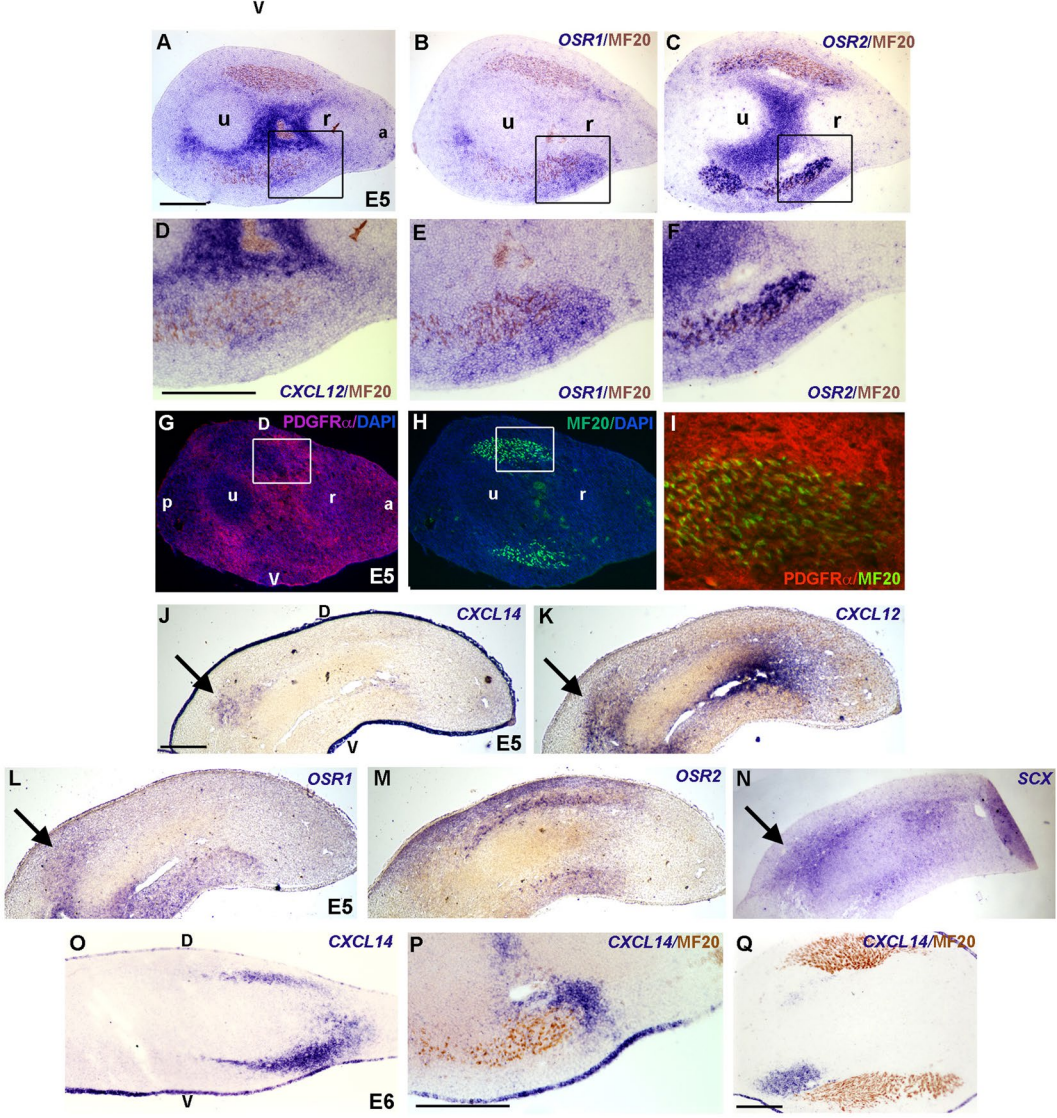
Chemokine expression in relation to CT markers in forelimb of E5 and E6 chick embryos. *In situ* hybridizations for *CXCL12* (**A**,**D**,**K**), *CXCL14* (**J**,**O**,**P**,**Q**), *OSR1* (**B**,**E**,**L**), *OSR2* (**C**,**F**,**M**) and immunohistochemical detections for PDGFRα (**G**,**I**), MF20 (**A**–**F**,**H**,**I**,**P**,**Q**) on serial transverse (**A**–**I**,**Q**) or longitudinal (**J**–**P**) sections. *CXCL12* is expressed in CT and MCT partially overlapping with *OSR1*, *OSR2* and PDGFRα expression. *CXCL14* exhibits a restricted expression in CT (**J**,**O**–**Q**), partially overlapping with *CXCL12*, *OSR1 and SCX* expression (arrows in **J**,**K**,**L**,**N**). (**D**,**E**,**F**,**I**) represent high magnifications of the squared regions respectively in (**A**,**B**,**C** and **G**,**H**,**D**): dorsal, V: ventral, a: anterior, p: posterior, r: radius, u: ulna. Bars: 200 µm in (**A**–**C**,**G**,**H**,**J**–**O**,**Q**); 100 µm in (**D**–**F**,**P**).

**Figure 2:**
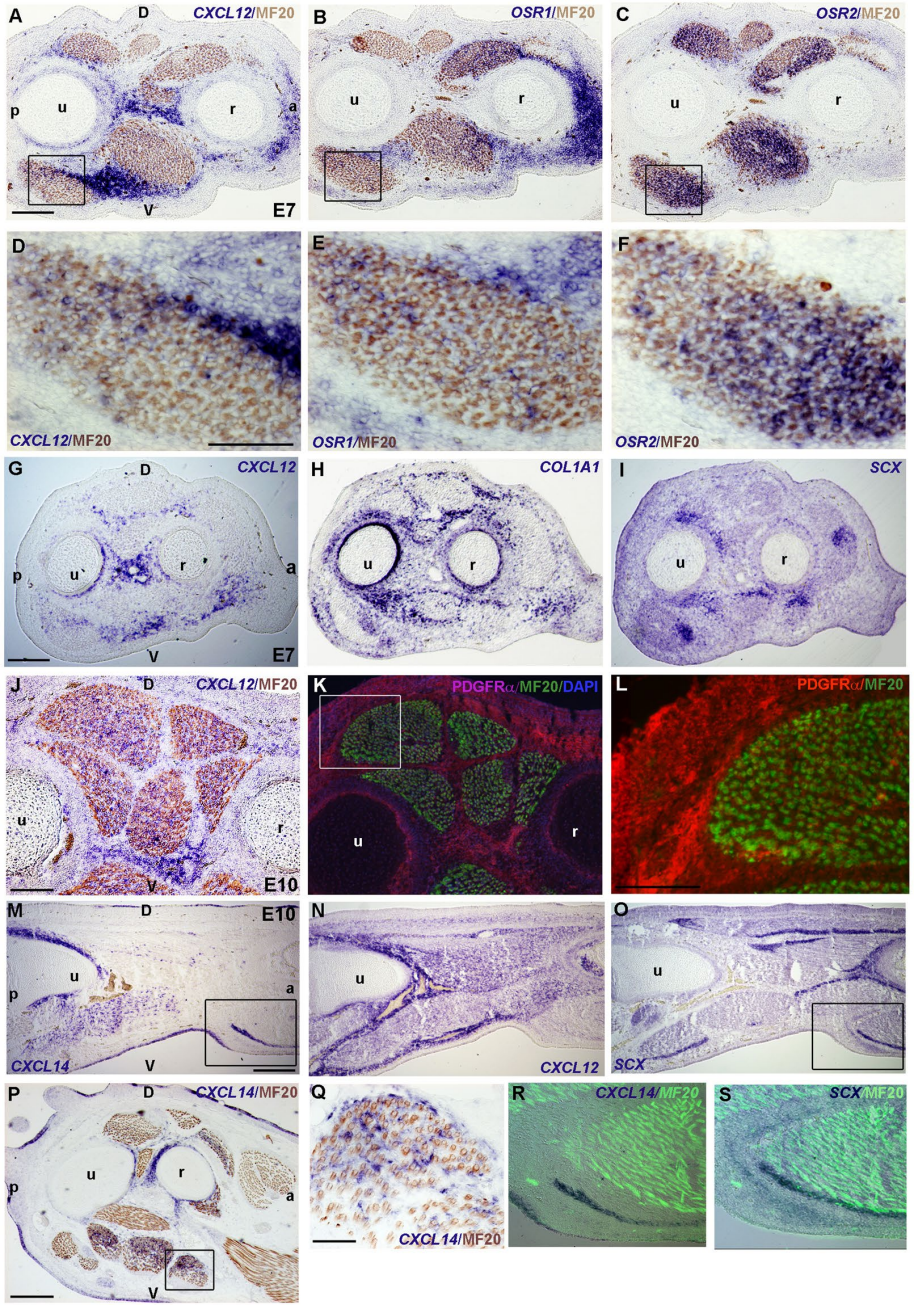
Chemokine expression in relation to CT markers in forelimb of chick embryos from E7 to E10. *In situ* hybridizations for *CXCL12* (**A**,**D**,**G**,**J**,**N**), *CXCL14* (**M**,**P**,**R**,**Q**), *OSR1* (**B**,**E**), *OSR2* (**C**,**F**), *COL1A1* (**H**), *SCX* (**I**,**O**,**S**) and immunohistochemical detections for PDGFRα (**K**,**L**), MF20 (**A**–**F**, **J**–**L**,**P**–**S**) on serial transverse (**A**–**L**, **P**,**Q**) or longitudinal (**M**–**O**,**R**,**S**) sections. *CXCL12* is expressed in CT and MCT partially overlapping with *OSR1*, *OSR2* and PDGFRα expression but with no evident colocalisation with *COL1A1* or *SCX* expression. *CXCL14* expression partially overlaps with *CXCL12* expression in CT and MCT and is observed in the vicinity of *SCX* expression in CT giving rise to tendon. (**D,E,F,L,R,S**) represent high magnifications of the squared regions respectively in (**A**,**B**,**C**,**K**,**N**,**O**). D: dorsal, V: ventral, a: anterior, p: posterior, r: radius, u: ulna. Bars: 200 µm in (**A**–**C**,**G**–**I**,**M**–**P**); 100 µm in (**D**–**F**,**J**,**K**); 50 µm in (**Q**–**S**).

We analysed the expression patterns of *CXCL12* and *CXCL14* in chick limbs at different developmental stages. Comparison was made with *OSR1*, *OSR2* and PDGFR-α, three ICT-associated markers and with *SCX* for tendon CT. Limb differentiated muscles were visualized by myosin expression (MF20 antibody). At E5 of development, *CXCL12* displayed a diffuse expression in limb CT, with a strong expression in ICT between cartilage elements, overlapping with *OSR2* expression (Fig. 1A,C). *CXCL12* was also expressed in MCT (Fig. 1D), overlapping with *OSR1* and *OSR2* expression (Fig. 1D–F). *CXCL12* also colocalised with PDGFR-α, which appeared widely expressed in the forelimb ICT and at a lower level in MCT (Fig. 1G–I), as previously described^43,44^. At E5, *CXCL14* was expressed in forelimb ectoderm and faintly in proximal CT (Fig. 1J, arrow), in a region where *CXCL12* was also expressed (Fig. 1K, arrow). *CXCL14* exhibited a partial overlap with *OSR1* and *SCX* expression but not with that of *OSR2* in proximal limb regions (Fig. 1J,L–N, arrows). From E6, in addition to ectoderm expression, *CXCL14* was expressed in a subpopulation of ICT mostly located in limb ventral regions (Fig. 1O–Q). At E7, *CXCL12* expression was still observed in ICT and MCT, overlapping with *OSR1* expression in ICT and both *OSR1* and *OSR2* expression in MCT (Fig. 2A–F). At this stage, *CXCL12* expression partially overlapped with *COL1A1* but not with *SCX* (Fig. 2G–I). At E10, *CXCL12* expression was reminiscent of that of PDGFR-α in ICT and MCT (Fig. 2J–L). In addition to ectodermal expression, *CXCL14* was observed in ICT surrounding cartilage (Fig. 2M), but also in MCT of a subgroup of ventral muscles (Fig. 2M,P) and in ICT at the vicinity of *SCX* expression (Fig. 2R,S). At this stage, *CXCL14* and *CXCL12* expression did not obviously overlapped in ICT, but partially colocalised around cartilage and in MCT (Fig. 2M,N).

Taken together, our results show that *CXCL12* and *CXCL14* chemokines exhibit distinct and dynamic expression patterns during chick limb development in ICT and MCT. *CXCL12* expression is widespread in limb ICT and MCT, while *CXCL14* is expressed in the vicinity of a subset of tendons and restricted to MCT of specific muscles, mostly located ventrally in limbs. The comparison of *CXCL12* and *CXCL14* expression with that of limb CT-markers shows a good correlation between the expression of *CXCL12* and *OSR* transcription factors, while *CXCL14* expression appears complementary in some limb regions to the tendon CT-marker *SCX*, suggesting a differential role for the two chemokines in limb CT regulation.

### *CXCL12* and *CXCL14* regulate the expression of different CT markers during chick limb development

**Figure 3:**
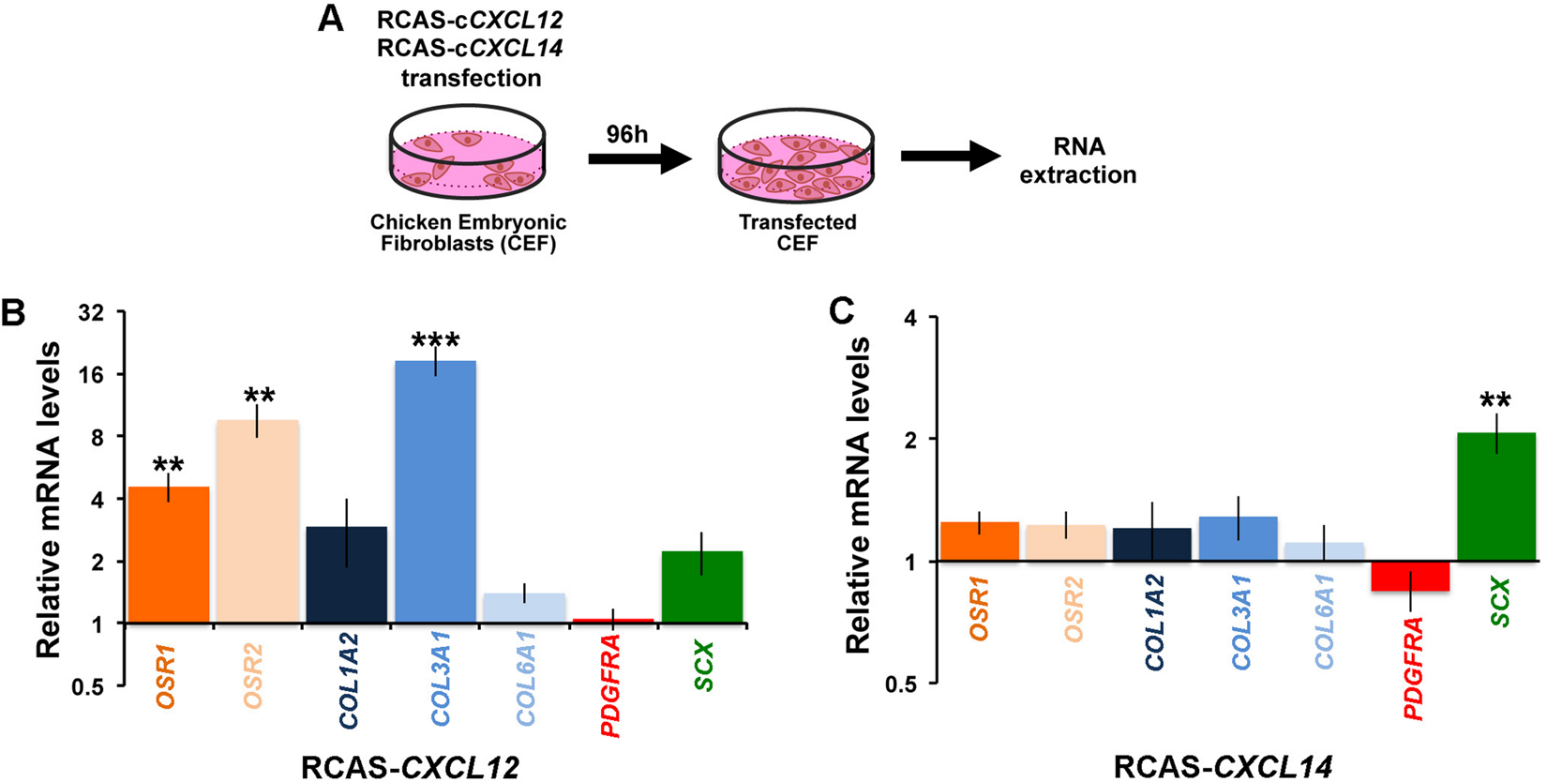
Overexpression of CXCL12 and CXCL14 chemokines increases the expression of CT markers *in vitro*. (**A**) Experimental scheme of *in vitro* transfection of chicken embryonic fibroblasts (CEF) by RCAS viruses expressing chick *CXCL12* or *CXCL14* constructs. (**B**,**C**) RT-qPCR analyses of the expression level of CT markers in CEF overexpressing *CXCL12* (B, n = 6) or *CXCL14* (C, n = 6) showing that *CXCL12* upregulates significantly *OSR1*, *OSR2* and *COL3A1*, while *CXCL14* increases *SCX*. For each gene, the mRNA levels of control cultures (n = 6) were normalised to 1. P values were analysed by two-tail and unpaired Student’s t-test using Microsoft Excel. **P < 0.01; ***P < 0.001; Error bars indicate s.d.

**Figure 4:**
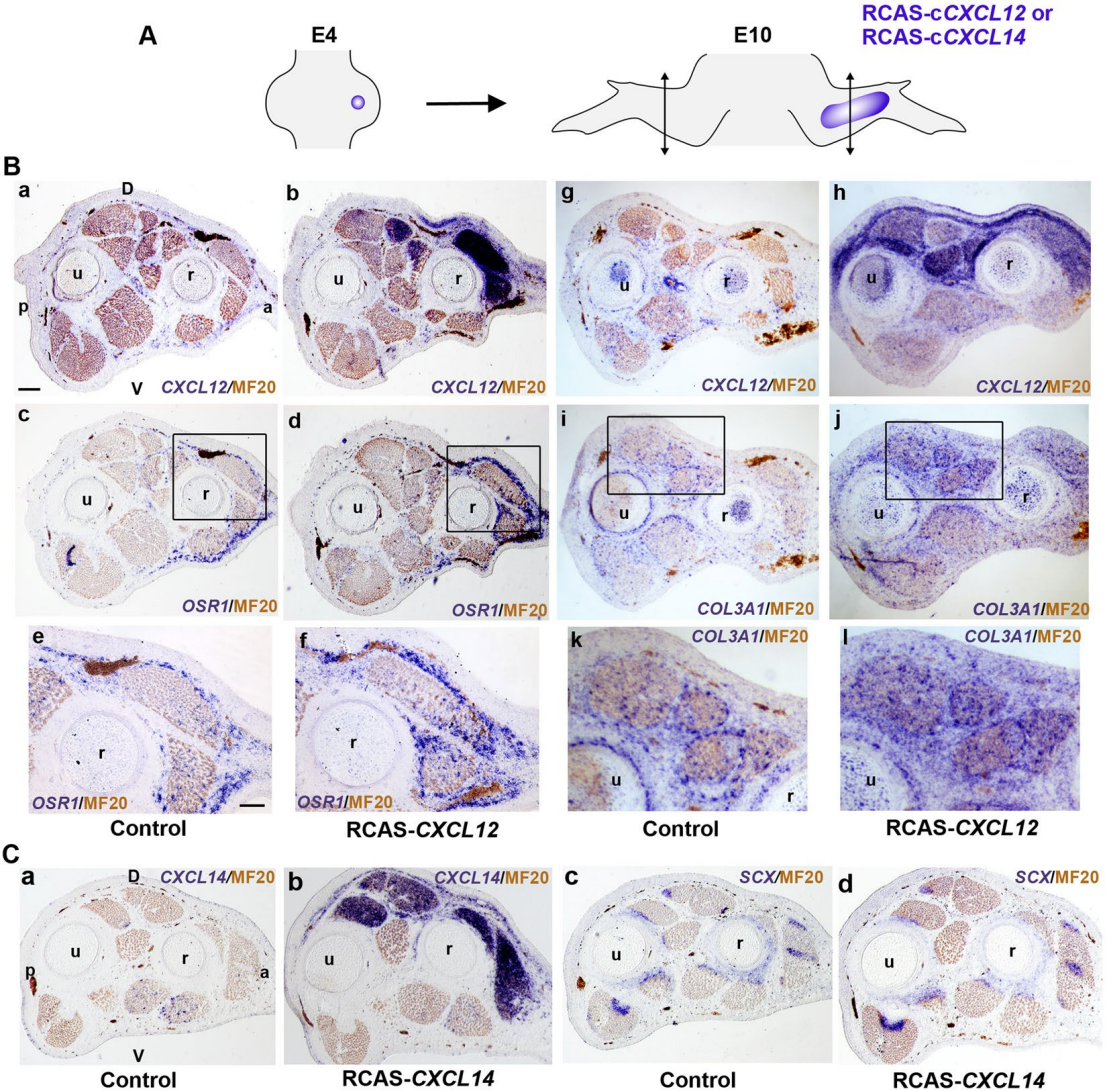
Overexpression of CXCL12 and CXCL14 chemokines *in vivo* increases the expression of CT markers in the chick embryonic forelimb. (**B**) Ectopic expression of *CXCL12* upregulates *OSR1* and *COL3A1* expression (n = 6). *In situ* hybridizations for *CXCL12* (**a**,**b**,**g**,**h**), *OSR1* (**c**–**f**) and *COL3A1* (**i**–**l**) on serial transverse sections of control (**a**,**c**,**e**,**g**,**i**,**k**) and grafted (**b**,**d**,**f**,**h**,**j**,**l**) E10 chick forelimbs. (**e**,**f**,**k**,**l**) represent high magnifications of the squared regions respectively in (**c**,**d**,**i**,**j**). (**C**): Ectopic expression of *CXCL14* does not modify *SCX* expression (n = 6). *In situ* hybridizations for *CXCL14* (**a**,**b**) and *SCX* (**c**,**d**) on serial transverse sections of control (**a**,**c**) and grafted (**b**,**d**) E10 chick forelimbs. (**D**) dorsal, V: ventral, a: anterior, p: posterior, r: radius, u: ulna. Bars: 200 µm in B (**a**-**d**, **g**–**j**), C (**a**–**d**); 100 µm in B (**e**,**f**,**k**,**l**).

To test the involvement of CXCL12 and CXCL14 in limb CT differentiation, we performed CXCL12 and CXCL14 gain-of-function experiments *in vitro* and *in vivo*. We first overexpressed CXCL12 and CXCL14 chemokines in primary cultures of E10 chick embryonic fibroblasts using the replication-competent RCAS retrovirus system. Chick embryonic fibroblasts were transfected with recombinant retroviruses that spread in dividing cells, allowing a general expression of the gene of interest after 4 days of culture (Fig. 3A). *CXCL12* overexpression in chick embryonic fibroblasts led to a significant increase in the mRNA levels of *OSR1*, *OSR2* and *COL3A1* genes, while the expression of *COL1A2, COL6A1, PDGFRA* and *SCX* was not significantly modified (Fig. 3B). *CXCL14* overexpression significantly increased the mRNA levels of the tendon marker *SCX*, but did not change the expression levels of the other CT-associated markers (Fig. 3C). We next overexpressed *CXCL12* and *CXCL14* in ovo, by grafting RCAS-*CXCL12* or RCAS-*CXCL14* producing fibroblasts into limb buds of E4 chick embryos to allow ectopic gene expression in limb regions^24^ (Fig. 4A). Embryos were collected at E10 and the expression of CT markers was analysed by *in situ* hybridization to transverse limb sections (Fig. 4A). Ectopic *CXCL12* expression (Fig. 4Bb,h) resulted in an increase of *OSR1* and *COL3A1* expression in the infected limb regions (Fig. 4Bd,f,j,l) compared to control limbs (Fig. 4B). We did not observe any obvious change of *OSR2* expression by *in situ* hybridization in the RCAS-*CXCL12*-infected regions (data not shown). *CXCL14* overexpression did not induce ectopic *SCX* expression in chick forelimbs when compared to control limbs (Fig. 4C), although we cannot exclude an increase of *SCX* expression in tendons.

Altogether, these results show that CXCL12 and CXCL14 chemokines differentially regulate CT-associated markers *in vivo* and *in vitro*. CXCL12 positively regulates the expression of the ICT markers *OSR1* and *COL3A*, while CXCL14 regulates the tendon marker *SCX*, at least *in vitro*.

### Misregulation of CXCL12/CXCR4 axis in limb alters vascular network

In order to investigate which receptor could be involved in the effect of CXCL12 on ICT gene expression, we first analysed the expression of the two recognized CXCL12 receptors, CXCR4 and CXCR7^38^, in developing chick limbs. At E5, CXCR4 was expressed in endothelial cells labelled with MEP21 (Supp. Fig. 1A–C), as already described in chick embryos^45,46^, while *CXCR7* was observed in limb ICT, with a strong expression in ICT surrounding cartilage elements (Supp. Figure 1A–C). At E10, *CXCR7* appeared expressed in some regions of ICT around muscles, while CXCR4 was still expressed in endothelial cells at this stage (Supp. Fig. 1D–F).

**Figure 5:**
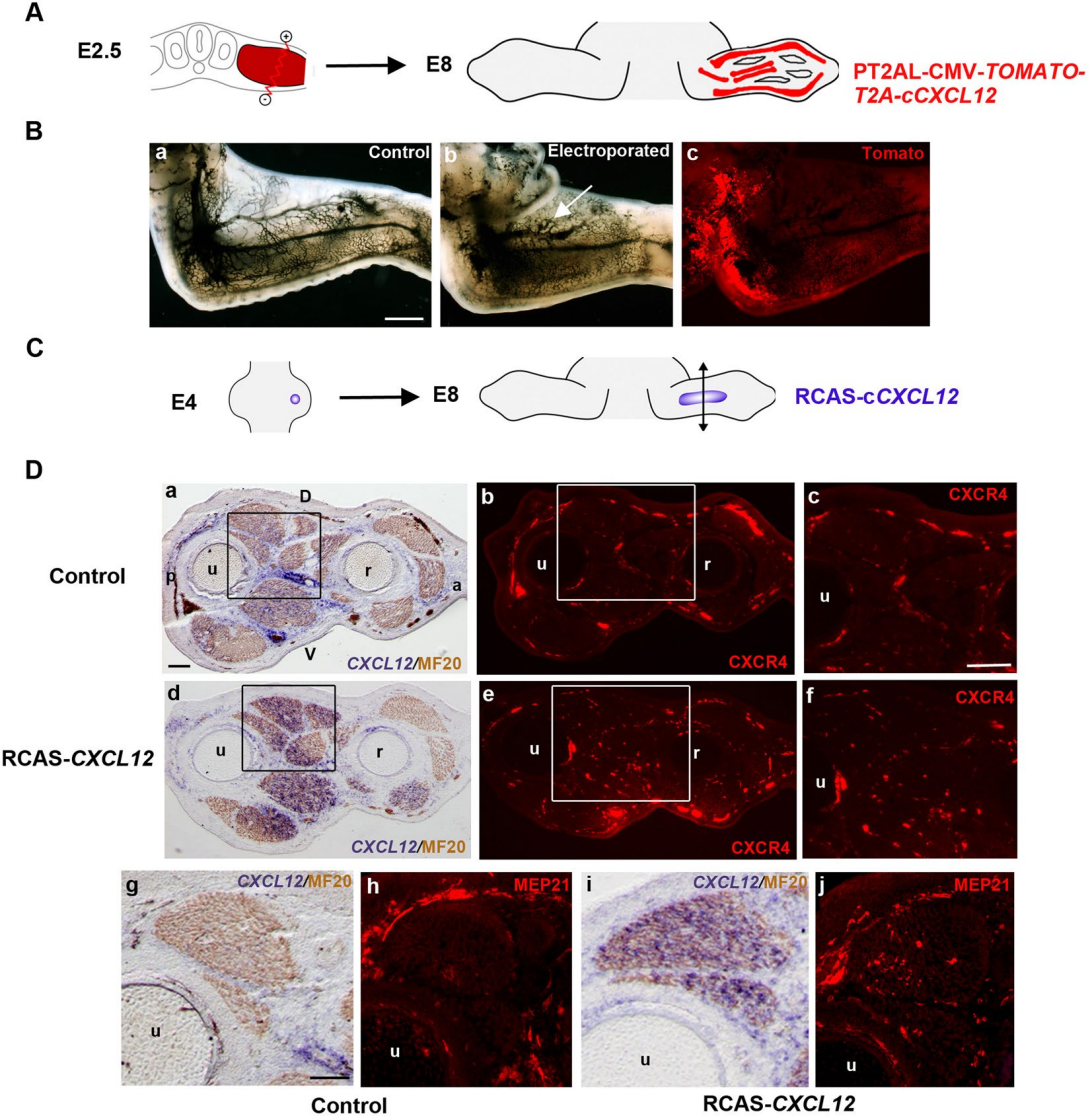
Overexpression of *CXCL12* affects limb vessel network and distribution of CXCR4-positive endothelial cells. (**A**) Experimental scheme of the electroporation procedure of PT2AL-CMV-*TOMATO-T2A-CXCL12* construct in the chick limb lateral plate mesoderm. (**B**) Ectopic expression of *CXCL12* in the lateral plate mesoderm resulted in abnormal limb vascular network in electroporated wings (**b**,**c**, n = 5/8) when compared to control wings (**a**, n = 8). (**a,b**) E8 wings after black ink injection. (**c**) Tomato expression in corresponding electroporated wing. (**C**) Experimental scheme of the grafting procedure of CEF pellets expressing chick *CXCL12* constructs in chick embryonic forelimbs. (**D**) Ectopic expression of *CXCL12* modifies the distribution of CXCR4- and MEP21-positive cells in chick limbs (n = 6). *In situ* hybridization for *CXCL12* (**a**,**d**,**g**,**i**) and immunochemistry for CXCR4 (**b**,**c**,**e**,**f**) and MEP21 (**h**,**j**) on serial transverse sections of control (**a**–**c**,**g**,**h**) and grafted (**d**–**f**,**I**,**j**) E8 chick forelimbs. (**c**,**f**) represent high magnifications of the squared regions respectively in (**b**,**e**). D dorsal, V: ventral, a: anterior, p: posterior, r: radius, u: ulna. Bars: 1 mm in B (**a**–**c**), 200 µm in D (**a**,**b**,**d**,**e**), 100 µm in D (**c**,**f**) 50 µm in D (**g**–**j**).

Consistent with the endogenous CXCR4 expression in endothelial cells of developing chick limbs (Supp. Fig. 1) and adults^47^, the CXCL12/CXCR4 axis is known to be involved in vasculogenesis during development and in pathological conditions^48,49^. We thus analysed the consequences of *CXCL12* overexpression for limb vascular network in chick embryos. *CXCL12* was overexpressed in limb CT by electroporating a PT2AL-CMV-*TOMATO-T2A-CXCL12* construct in chick limb lateral plate mesoderm (Fig. 5A) using the Peptide 2 A system allowing simultaneous expression of Tomato and the gene-of -interest^50^. Limb lateral plate mesoderm was electroporated at E2.5 and embryos were collected at E8 for ink injection in the heart. As expected, *CXCL12* overexpression in limb CT visualized with Tomato fluorescence affected the vascular network in electroporated wings (Fig. 5Bb,c), when compared to controls (Fig. 5Ba). The vascular phenotype was characterized by a more or less enlargement of limb vessels and abnormal vascular branching upon CXCL12 overexpression (Fig. 5Bb,c). We next investigated the distribution of endothelial cells after CXCL12 overexpression, by analysing CXCR4 and MEP21 expression. Retroviral overexpression of *CXCL12* (Fig. 5C) modified the distribution of CXCR4- and MEP21-positive cells in the infected limb regions (Fig. 5Dd–f,i,j) compared to control limbs (Fig. 5Da–c,g,h), confirming that increased *CXCL12* expression in ICT resulted in an abnormal vascularisation in chick limbs.

To test the possible involvement of CXCR7 in the CXCL12 effect on CT-associated genes, we overexpressed a dominant-negative form of *CXCR7* (*dn-CXCR7*) in ovo, by grafting RCAS-*dn-CXCR7* producing fibroblasts into limb buds of E4 chick embryos. Embryos were collected at E10 to analyse the expression of CT markers by *in situ* hybridization to transverse limb sections. Ectopic *dn-CXCR7* expression (Supp. Fig. 2B,E,H,K) did not modify the expression of the transcription factors *OSR1*, *OSR2* and *SCX* nor that of *COL3A1* gene in infected limb regions (Supp. Fig. 2C,F,I,L) compared to controls (Supp. Fig. 2A,G,J). This indicates that CXCR7 receptor does not mediate the CXCL12 effect on the expression of CT markers.

Taken together, these data suggest that CXCL12 effect on ICT gene expression involves CXCR4 but not CXCR7 receptors.

### *CXCL12* and *CXCL14* expression is not regulated by FGF4

During development, FGF4 positively regulates *SCX* expression^24,51^ and the expression of ICT collagens, including *COL1A1, COL3A1* and *COL6A1* ^25^ in chick limbs. Interestingly, FGF has been shown to be involved in fibrosis regulation in different pathological situations^27^. We then analysed the effects of FGF4 overexpression on *CXCL12* and *CXCL14* expression in chick limbs. FGF4 beads were implanted into limbs at E4.5 and fixed 48 hours later (Supp. Figure 3A). As previously shown^24,25,51^, *SCX* expression was strongly induced at the vicinity of FGF4 beads in grafted limbs compared to normal *SCX* expression in control limbs (Supp. Fig. 3Ba,b,g,h). However, no ectopic expression of *CXCL12* and *CXCL14* was observed in ectopic *SCX* expression domains (Supp. Fig. 3Ba–f). In addition, we did not observe any *OSR1* and *OSR2* activation around FGF4 beads (Supp. Fig. 3Bg–l). However, an inhibition of *OSR1* and *OSR2* expression was observed in the vicinity of FGF4 beads (Supp.Fig. 3Bh,j,l) compared to control limbs (Supp. Fig. 5Bg,i,k). These results show that ectopic expression of FGF4 does not modify *CXCL12* and *CXCL14* expression but leads to the inactivation of both *OSR1* and *OSR2* genes in chick limbs, while increasing *SCX* expression. It is likely that *OSR* gene downregulation observed after FGF4 bead grafts is independent of CXCL12 and CXCL14 chemokines. However, since *OSR1* and *OSR2* overexpression inhibits *SCX* expression in chick limb cells^22^, these results suggest that *SCX* and *OSR* genes mutually repress each other in mesenchymal cells during chick limb development.

### The anti-fibrotic BMP signalling pathway negatively regulates the expression of *CXCL12* and *CXCL14*

**Figure 6:**
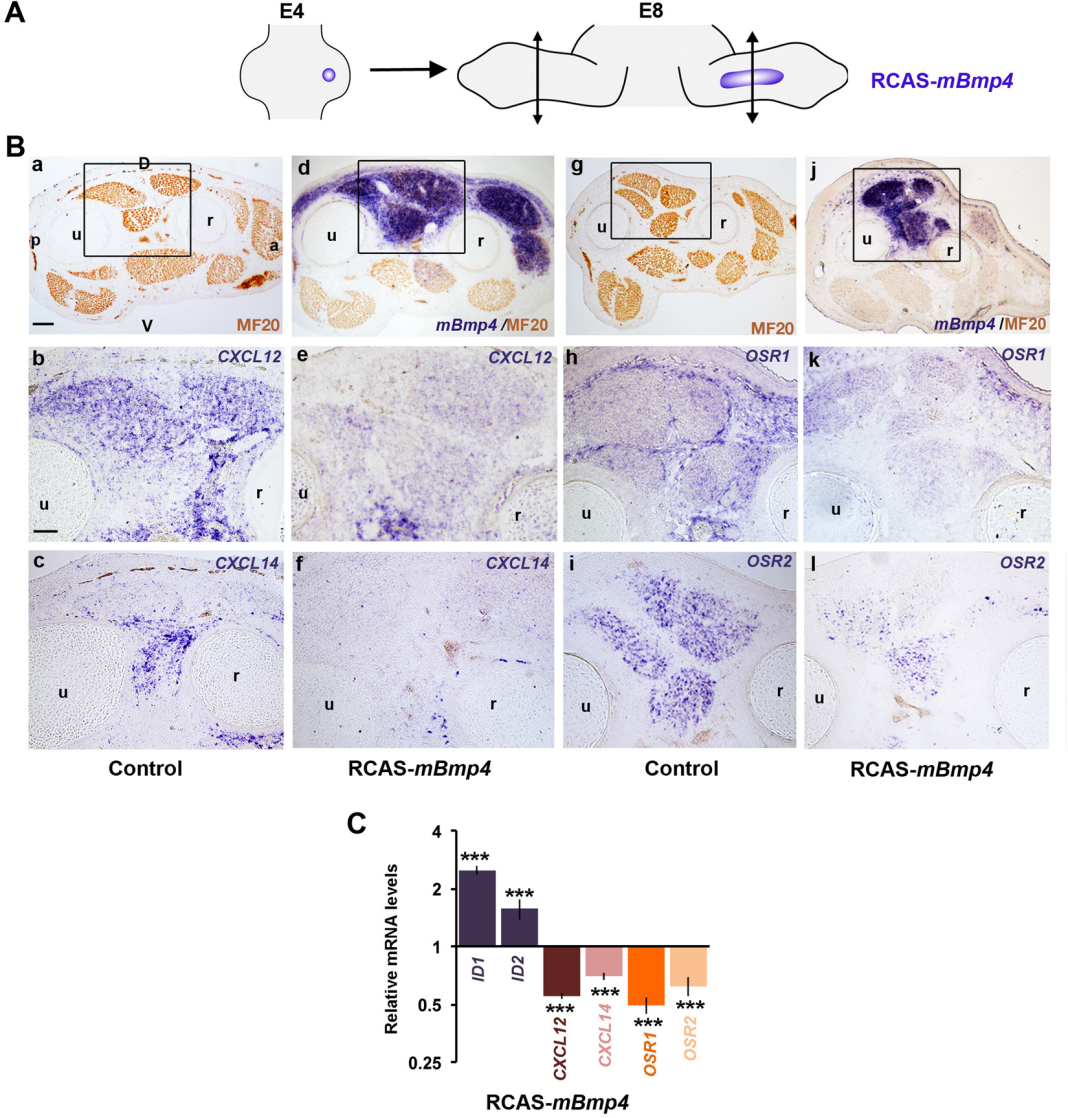
BMP signaling pathway down-regulates the expression of chick CT markers *in vivo* and *in vitro*. BMP signalling represses *CXCL12, CXCL14, OSR1*, and *OSR2* expression in the chick forelimb CT and MCT (n = 5). (**A**) Experimental scheme of the grafting procedure of CEF pellets expressing mouse *Bmp4* construct in the chick embryonic forelimb. (**B**) *In situ* hybridizations for *CXCL12* (**b**,**e**), *CXCL14* (**c**,**f**), *OSR1* (**h**,**k**), *OSR2* (**i**,**l)**, *mBmp4* (**d**,**j**) and immunohistochemical detection for MF20 (**a**,**d**,**g**,**j**) on serial transverse sections of control (**a**–**c**,**g**–**i**) and grafted (**d**–**f**,**j**–**l**) E8 chick forelimbs. The squared regions in (**a**,**d**,**g**,**j**) delineate the high-magnified parts of the adjacent sections revealed for chemokines or CT markers. D: dorsal, V: ventral, a: anterior, p: posterior, r: radius, u: ulna. Bars: 200 µm in B (**a**,**d**,**g**,**j**), 100 µm in B (**b**,**c**,**e**,**f**,**h**,**k**,**i**,**l**). (**C**): RT-qPCR analyses of expression level of readout targets of BMP4 signalling and of CT markers in CEF expressing RCAS-*mBmp4* construct, showing that BMP4 expression significantly decreases *CXCL12, CXCL14, OSR1* and *OSR2* expression (n = 6). For each gene, the mRNA levels of control cultures (n = 6) were normalised to 1. P values were analysed by two-tail and unpaired Student’s t-test using Microsoft Excel. ***P < 0.001; Error bars indicate s.d.

**Figure 7:**
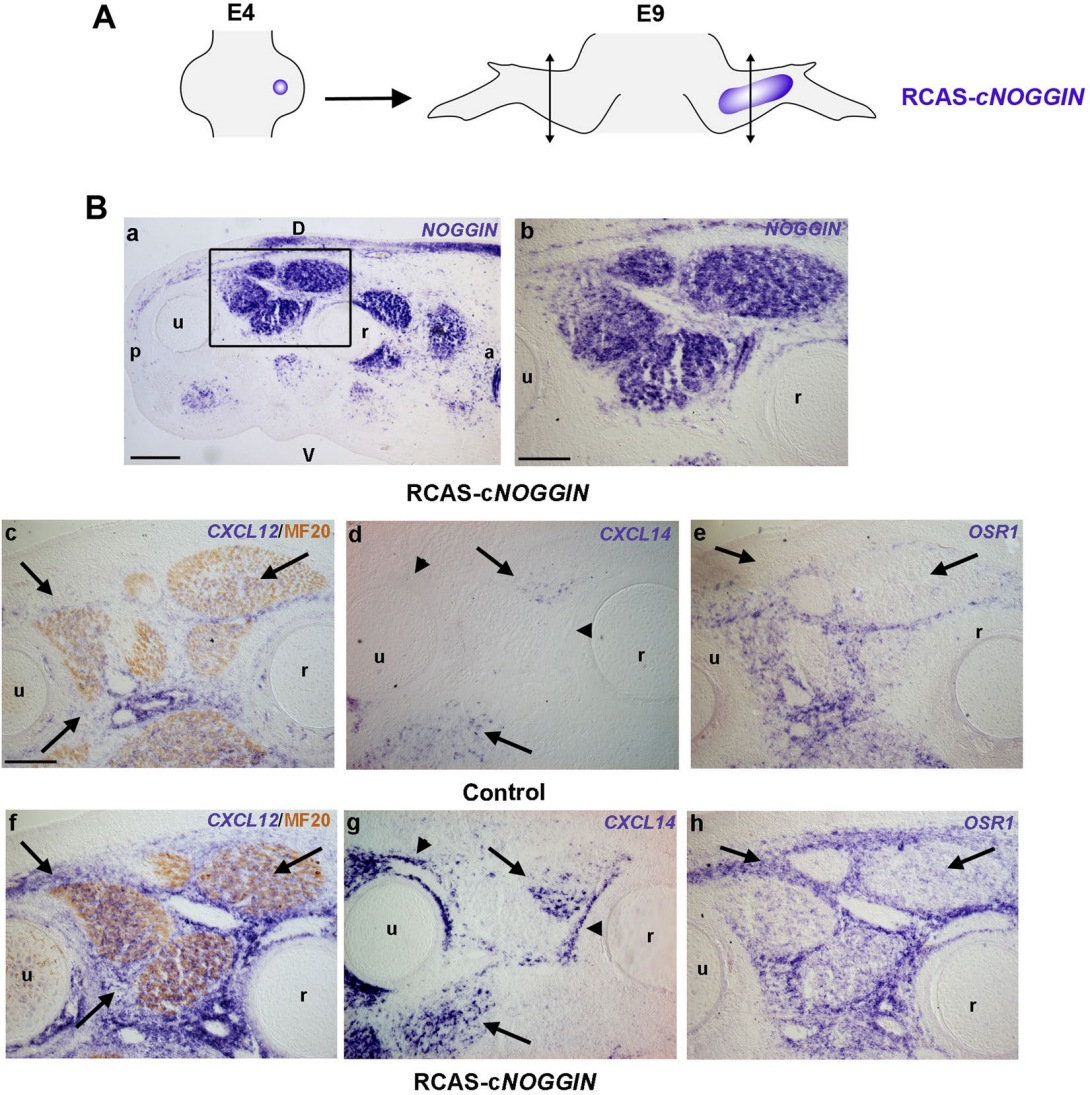
Inhibition of BMP signalling activates the expression of chick CT markers *in vivo*. BMP inhibition increases *CXCL12, CXCL14* and *OSR1* expression in the chick forelimb CT and MCT (n = 4). (**A**) Experimental scheme of the grafting procedure of CEF pellets expressing chick *NOGGIN* construct in the chick embryonic forelimb. (**B**) *In situ* hybridization for *NOGGIN* (**a**,**b**), *CXCL12* (**c**,**f**), *CXCL14* (**d**,**g**), *OSR1* (**e**,**h**) and immunohistochemical detection for MF20 (**c**,**f**) on serial transverse sections of control (**c**–**e**) and grafted (**f**–**h**) E9 chick forelimbs. The squared regions in a delineate the high-magnified region of the adjacent sections revealed for *NOGGIN*, chemokines or CT markers. Arrows indicate the expression in CT and MCT. Arrowheads indicate the expression in perichondrium. D: dorsal, V: ventral, a: anterior, p: posterior, r: radius, u: ulna. Bars: 200 µm in A (**b**–**h**), 100 µm in A (**a**).

BMP signalling has been demonstrated as an antagonist of fibrosis in various organs^52^ but very few studies have investigated its potential role in the regulation of CT types during development. Conversely to FGF4, BMP4 inhibits *SCX* expression in chick embryonic limbs^12,53^. We then looked at the possible effect of BMP signalling on CXCL12 and CXCL14 expression *in vivo* by overexpressing BMP4 in chick limbs. Pellets of transfected RCAS-*mBmp4* fibroblasts were grafted into E4 limbs, which were collected at E8 to analyse the expression of chemokines and CT markers (Fig. 6A). *Bmp4* overexpression resulted in a downregulation of *CXCL12* and *CXCL14* expression (Fig. 6Bd–f) and a concomitant decrease in both *OSR1* and *OSR2* expression in limb mesenchyme (Fig. 6Bj–l), compared to control (Fig. 6Ba–c,g–i). These results were supported *in vitro* by the analysis of mRNA levels of chemokines and CT markers in embryonic fibroblasts overexpressing *Bmp4*, which showed downregulation of *CXCL12*, *CXCL14*, *OSR1* and *OSR2* expression (Fig. 6C). These data evidence that BMP4 gain-of-function experiments lead to a decrease of chemokine and CT marker expression in chick limb cells. In order to test if BMP loss-of-function experiments would provoke the opposite result, BMP signalling pathway was inhibited *in vivo* by overexpressing the BMP antagonist Noggin in chick limbs^54^. Pellets of transfected RCAS-*cNOGGIN* producing fibroblasts were grafted into E4 limbs, which were collected at E9 to analyse the expression of chemokines and CT markers (Fig. 7A). *NOGGIN* overexpression led to an upregulation of *CXCL12* and *CXCL14* expression in both ICT and MCT (Fig. 7Ba,b,f,g) and a concomitant increase in *OSR1* expression (Fig. 7Bh), compared to control limbs (Fig. 7Bc–e). In this experimental design, *OSR2* and *SCX* expression was not obviously modified (data not shown).

Taken together, these data show that the anti-fibrotic factor BMP4 negatively regulates the expression of *CXCL12* and *CXCL14* chemokines and *OSR1* and *OSR2* transcription factors, while BMP inhibition is sufficient to induce the expression of *CXCL12*, *CXCL14* and *OSR1* in chick limbs.

### Mechanical forces act upstream CXCL12 and CXCL14 to regulate CT markers

**Figure 8:**
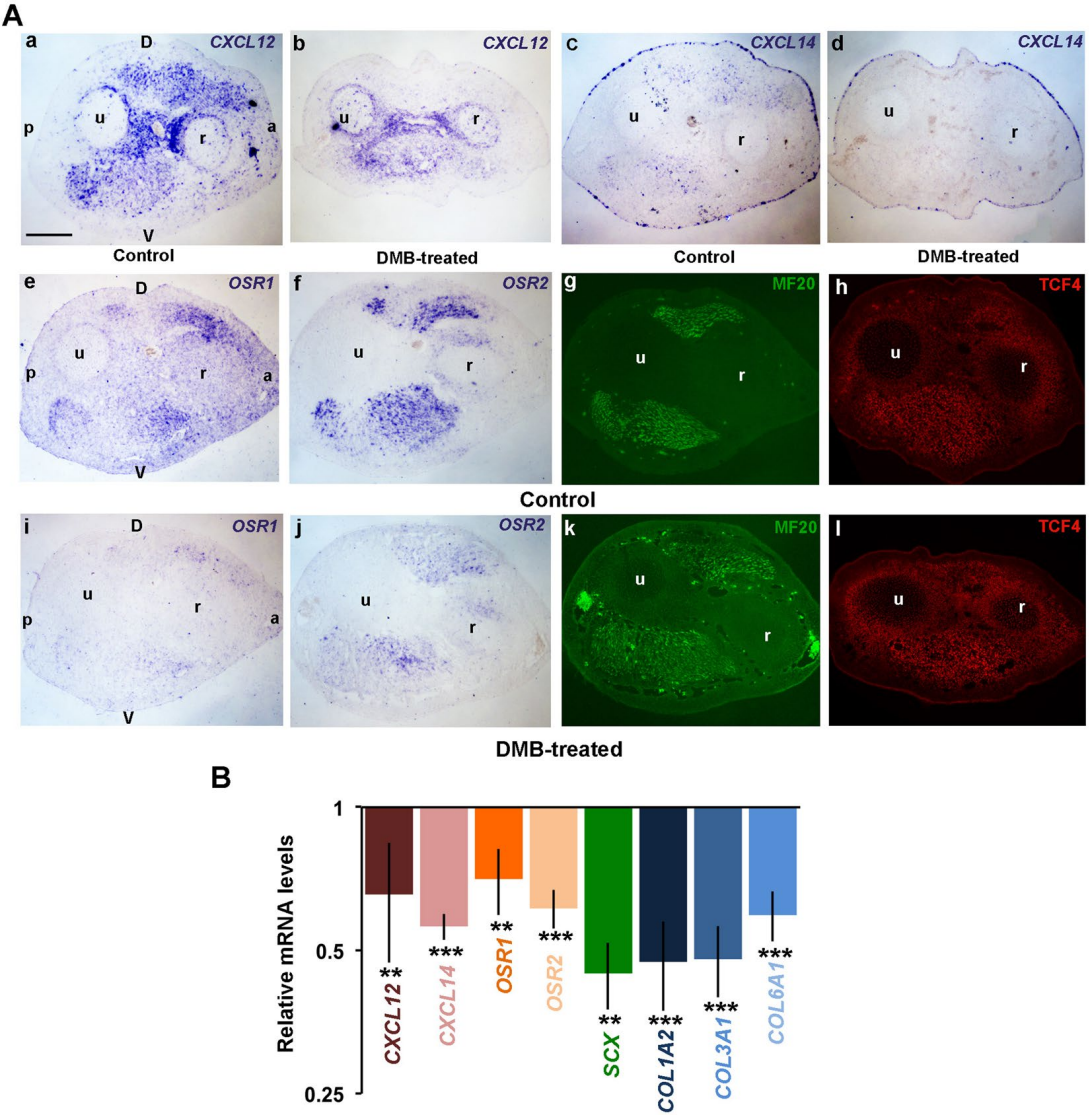
Mechanical forces act upstream CXCL12 and CXCL14 chemokines to regulate the expression of CT markers in the chick embryonic forelimb. (**A**) Immobilisation of the chick embryonic forelimb downregulates the expression of CXCL12 and CXCL14 and of the CT markers (n = 3). *In situ* hybridizations for *CXCL12* (**a**,**b**), *CXCL14* (**c**,**d**), *OSR1* (**e**,**i**), *OSR2* (**f**,**j**) and immunodetection of MF20 (**g**,**k**) and TCF4 (**h**,**l**) on serial transverse sections of control (**a**,**c**,**e**–**h**) and DMB-treated (**b**,**d**,**i**–**l**) E6.5 chick forelimbs. (**D**) dorsal, V: ventral, a: anterior, p: posterior, r: radius, u: ulna. (**B**) RT-qPCR analyses of *CXCL12*, *CXCL14* and CT markers in E7.5 immobilized chick forelimbs (n = 4) showing that both chemokine and CT marker expressions are reduced in immobilisation conditions. For each gene, the mRNA levels of control limbs (n = 4) were normalized to 1. P values were analyzed by two-tail and unpaired Student’s t-test using Microsoft Excel. **P < 0.01; ***P < 0.001; Error bars indicate s.d.

The musculoskeletal system is sensitive to mechanical loads exerted by muscle contractions. Mechanical forces generated by muscle activity have been shown to be crucial for the formation of components of the musculoskeletal system during development^51,55,56^. In the adult, studies indicate that mechanical forces contribute to fibrosis by regulating CT fibroblasts in bone, cartilage and interstitial tissues of most organs^57^. During development, *SCX* expression is sensitive to mechanical forces in tendons of mouse and chick limbs^51,58^ but no investigation has been conducted concerning the role of mechanical signals on ICT and MCT during development. To assess the effect of mechanical forces in this context, chick embryos were immobilised using the decamethonium-bromide (DMB) pharmacological agent, an acetylcholine agonist leading to rigid muscle paralysis and immobilisation of the embryo^59^. DMB or control solutions were applied in E4.5 embryos and limbs were collected 2 or 3 days later to analyse the expression of chemokines and CT marker by *in situ* hybridization and RT-qPCR. DMB has been shown to bind motor endplates and block muscle contraction^60^. As neuromuscular contractions in chick embryonic limbs are effective from E5.5^61^, limb muscles were not grossly affected at E6.5 after 2 days of immobilisation (Fig. 8Ah, l), while paralysis leads to muscle degeneration later during development, as previously described^62^. *CXCL12* and *CXCL14* expression was reduced in MCT and ICT surrounding muscles in paralysed limbs (Fig. 8Ab,d). However, *CXCL12* expression was maintained in CT surrounding bones, while *CXCL14* was still expressed in the ectoderm in immobilised conditions (Fig. 8Ab,d). The expression of *OSR1* and *OSR2* was also decreased in the absence of mechanical activity compared to control limbs (Fig. 8Ae–j). RT-qPCR analyses confirmed the downregulation of the mRNA levels of *CXCL12, CXCL14, OSR1* and *OSR2* in paralysed limbs (Fig. 8B) and also showed that the mRNA levels of *COL1A2*, *COL3A1* and *COL6A1* genes were significantly reduced in immobilised conditions. The decrease of CT marker expression cannot be attributed to a loss of CT cells since TCF4-positive cells were still observed in limbs of immobilised as compared to control embryos (Fig. 8Ah,l). Taken together, these results show that the expression of *CXCL12*, *CXCL14* and CT markers is sensitive to mechanical forces during limb development.

## Discussion

Fibrosis is a pathological reaction of CT arising from chronic inflammation, which progressively destroys tissue architecture. It results from the activation of fibroblasts, which produces excessive deposition of extracellular matrix. Chemokines appear to be central mediators of fibrosis initiation and progression^63^. During development, embryonic fibroblasts are constantly activated to participate to tissue remodelling^64^. Therefore, understanding fibroblast features and regulation during development is an important issue to address fibrosis mechanisms. In this study, we show that CXCL12 and CXCL14 chemokines positively regulate the expression of different CT-associated markers in chick limb fibroblasts. In addition, we show that BMP signalling, known for its anti-fibrotic role, negatively regulates *CXCL12* and *CXCL14* expression in limb CT during development. Finally, mechanical forces, which are essential for the progression of fibrosis^65^, also regulate the expression of *CXCL12* and *CXCL14* and CT markers.

We show here that the chemokine CXCL12 is sufficient to activate the expression of ICT markers, *OSR1* and *COL3A1* in chick limb fibroblasts *in vitro* and *in vivo*, identifying CXCL12 as a promoting factor of ICT differentiation during chick limb development. We show that, although expressed in some regions of the limb ICT, CXCR7 receptor is not involved in the CXCL12 effect on the expression of CT markers. *CXCL12* overexpression in chick limb ICT modifies the distribution of limb endothelial cells expressing CXCR4 receptor and leads to abnormalities in limb vasculogenesis, mostly characterized by the enlargement of some vessels and aberrant vascular branching. Interestingly, CXCL12 has been shown to promote PDGF-B production via direct activation of transcription of *Pdgfb* gene in endothelial cells^66^. PDGF-B is known to play a role in CT remodelling and fibrosis^67,68^ and to induce collagen transcription via PDGFR-α expressed in CT in chick limbs^44^. Consequently, it can be hypothesized that increasing CXCL12/CXCR4 signalling pathway in chick embryonic limbs modifies the limb vascular network and the secretion of PDGF-B which, in turn, would promote the expression of ICT markers (Supp. Fig. 4). The promoting effect of CXCL12 on ICT during development is consistent with the recognized role of CXCL12/CXCR4 axis in adult fibrosis. *CXCL12* expression is increased in different human fibrotic pathological situations, such as idiopathic pulmonary fibrosis^69^ and chronic pancreatitis^70^. In human lung and prostate fibroblasts, CXCL12 has been shown to induce, via the CXCR4 receptor, the expression of α-SMA, the key marker of activated fibroblast^71,72^.

As CXCL12, CXCL14 has been demonstrated as being involved in tissue fibrosis. CXCL14 expression is upregulated in idiopathic pulmonary fibrosis^73^ and chronic inflammatory arthritis^74^ and has been shown to stimulate prostate fibroblast proliferation and migration^75^. Our results show that CXCL14 specifically activates the tendon-CT marker *SCX* in chick fibroblasts, while not affecting the other CT markers. It has been shown recently that SCX controls fibroblast activation in the heart and could be a potent regulator in fibrotic diseases in the cardiovascular system^18^. In addition, *SCX* is activated by the pro-fibrotic factor TGF-β during chick or mouse limb development^51,76^. Previous experiments have shown that limb ectoderm induces *SCX* expression in limb mesenchyme at E3.5 but is not required for *SCX* maintenance after E4.5 of development^12^. As *CXCL14* is expressed in limb ectoderm (Fig. 2,^30^), one attractive hypothesis would be that the ectodermal-derived *CXCL14* regulates *SCX* expression in limb mesenchymal cells in early limb buds.

We show here that forced-expression of BMP4 decreases *CXCL12*, *CXCL14*, *OSR1* and *OSR2* expression in embryonic fibroblasts and limbs, while the inhibition of BMP signalling by *NOGGIN* overexpression leads to increased expression of *CXCL12*, *CXCL14* and *OSR1* in chick limbs. The anti-fibrotic effects of BMPs has been demonstrated in many organs, where it has been shown that BMP acts by inhibiting the pro-fibrotic activity of TGFβ or directly repressing the expression of *Ctgf* gene, encoding a pro-fibrotic factor, and *Acta2* gene, encoding α-SMA^52^. Because *CXCL12* positively regulates *OSR1* expression (Fig. 4), it is tempting to speculate that the modification of *OSR1* expression by BMP signalling is a consequence of *CXCL12* down- and upregulation following BMP gain- and loss-of-function experiments, respectively. This would indicate a pivotal role for CXCL12 and CXCL14 chemokines as downstream targets of BMP4 signalling pathway to regulate ICT markers during chick limb development (Supp. Figure 4). In bone marrow stromal cell lines, SMAD-binding elements have been identified in the *Cxcl12* promoter and BMP4 has been shown to regulate *Cxcl12* expression, as treatment of stromal cells with the BMP antagonist Noggin significantly increased *Cxcl12* levels^77^. We show here that BMP overexpression or inhibition decreases or increases, respectively, *CXCL14* expression. As *SCX* expression in chick limbs has been shown to be restricted by localized BMP signalling^12^, it can be suggested that this restriction could occur through the inhibition of CXCL14 by BMP signalling.

The expression of *CXCL12* and *CXCL14* chemokines and their CT target genes is decreased in immobilisation conditions. Numerous studies have underlined the role of the mechanical activity as a profibrotic stimulus^65^. TGF-β, one important factor in fibrosis, is activated as the direct result of mechanical tension induced by the extracellular matrix^78^. Generation of mechanical tension results in the expression of α-SMA and the increase in matrix deposition in multiple tissues^65^. Mechanical activity has been also shown to modulate or synergize with *Scx* to promote the differentiation of mesenchymal cells towards tendon CT^79,80^. During chick embryonic development, muscleless or aneural limbs deprived of musculoskeletal activity exhibit reduced *SCX* expression^24^ and embryo immobilisation results in a FGF4-dependent downregulation of *SCX* in limbs^51^. The downregulation of *CXCL12* and *CXCL14* expression in immobilisation conditions is specific to MCT and ICT surrounding muscles, while not affecting that of cartilage regions (CXCL12) and ectoderm (CXCL14). Among the mechanisms involved in the response of adult muscle to exercise is the activation of the fibrotic TGF-β signalling pathway, which leads to an increase in the expression of CTGF and collagens in MCT^81^. TGF-β has been shown to control dense regular CT differentiation during limb development^82^, a mechanism regulated by mechanical activity^51^. Similar regulation could occur during development, as CXCL12 induces CTGF expression in fibroblasts^71^ and TGF-β has been shown to cooperate with CXCL12 in carcinoma-associated fibroblasts to induce α-SMA expression^83^. One possibility could be that TGF-β, dowstream of mechanical signals, interacts with CXCL12 to regulate ICT-associated genes in the limb, among which *CTGF* and *OSRs*.

Our study demonstrates for the first time that CXCL12 and CXCL14 chemokines differentially regulate the expression of specific CT genes, participating to the orchestration of ICT, MCT and dense regular CT differentiation during chick limb development (Supp. Figure 4). The developmental role for CXCL12 and CXCL14 chemokines in CT differentiation is fully consistent with their recognised functions in adult fibrosis processes, such as in chronic fibrotic pathologies^69,70,73,74^ and cancer-induced fibrosis^84^. CXCL12 and CXCL14 regulate the expression of CT-associated transcription factors in chick limb and are themselves controlled by the anti-fibrotic factor BMP and by the fibrotic properties of mechanical forces of the musculoskeletal system. Such an unexpected role of these chemokines during CT differentiation can contribute to a better understanding of the fibrosis mechanisms in adult pathological conditions.

## Methods

### Chick embryos

Fertilized chick eggs from commercial sources (JA 57 strain, Institut de Sélection Animale, Lyon, France, and White Leghorn, HAAS, Strasbourg) were incubated at 38 °C in a humidified incubator until appropriate stages. Embryos were staged according to the number of days in ovo (E). All experiments on chick live embryos have been realized before E14 and consequently are not submitted to a licensing committee, in accordance to the European guidelines and regulations.

### Constructs

The chicken *CXCL12*, *CXCL14* and *dnCXCR7* coding regions were amplified by PCR from an RT-PCR-derived cDNA library made from E5 chick limb, using primers containing ClaI restriction site. The *dnCXCR7* coding region is a truncated form of *CXCR7* lacking the C-terminal part of the sequence (Ray *et al*., 2012). *CXCL12*, *CXCL14* and *dnCXCR7* amplified sequence were then inserted into pCR-II TOPO vector using TOPO-TA cloning kit (Invitrogen). Inserted sequences were excised by digestion with ClaI and inserted into the ClaI site of replication-competent retroviral vector RCASBP(A)^85^. Clones containing the *CXCL12*, *CXCL14* or *dnCXCR7* coding regions in the sense orientation were selected. Mouse RCAS-*mBmp4* and chick RCAS-*cNOGGIN* constructs were described previously^86,87^.

### Production and grafting of recombinant/RCAS-expressing or control RCAS-expressing cells

Cells expressing RCAS-*cCXCL12*, RCAS-*cCXCL14*, RCAS-*mBmp4* or RCAS-*cNOGGIN* and control cells were prepared for grafting as previously described^54,88^. Primary fibroblasts from E10 chick embryos were plated *in vitro*, transfected with the RCAS constructs and grown until 90% confluence. Transfected fibroblasts were then transferred to uncoated culture dishes to allow cell pellet formation before grafting. Pellets of approximately 50 μm in diameter were grafted into the right wing bud of E4 chick embryos. Embryos were harvested at various times after grafting and processed for *in situ* hybridization or immunohistochemistry to tissue sections. The left wing was used as an internal control. Owing to certain variability in the virus spread among embryos, the expression of the ectopic gene was systematically checked by *in situ* hybridization.

### Lateral plate mesoderm electroporation

E2.5 chick embryos were electroporated as previously described^50^. PT2AL-CMV-*TOMATO- T2A-CXCL12* (1,5–2 μg/μl) construct was mixed with the transposase vector CMV-T2TP (molar ratio 1/3) to allow stable integration of genes in the chick genome, in a solution containing 0.33% carboxymethyl cellulose, 1% Fast green, 1 mM MgCl_2_ in PBS. DNA mix was injected with a glass capillary in the coelomic cavity between somatopleural and splanchnopleural mesoderm, at the level of the forelimb territory. Homemade platinum electrodes were placed above and bellow the embryo, with the negative electrode inserted into the yolk and the positive electrode localized above the presumptive forelimb region. Electroporation was delivered using a Nepagene NEPA21 electroporator with the following parameters: 2 pulses of 70 V, 1ms duration with 100 ms interpulse interval followed by 5 pulses of 40 V, 2ms duration with 500 ms interpulse interval. Electroporated embryos were harvested 6 days later and indian ink was injected into the heart using a glass capillary, in order to stain vessel organization.

### Bead implantation in chick limb buds

Heparin beads (Sigma) were soaked in 1 mg/ml of recombinant human FGF4 (R&D Systems) for 30 min on ice. FGF4 beads were grafted into the right wings of chick embryos at E4.5 and embryos were harvested 24 or 48 hours after grafting. Grafted right and contralateral left limbs were processed for *in situ* hybridization to sections.

### Drug administration in ovo

The stock solution for decamethonium bromide (DMB, Sigma D1260) was prepared at 10% dilution in Hank’s solution (Sigma H9269). The DMB solution was freshly prepared before each experiment at 0.5% in Hank’s solution with 1% of Penicillin-Streptomycin (Gibco, 15140). 100 µl of the DMB or control solution (Hank’s solution with 1% of Penicillin-Streptomycin) were administrated in ovo at E4 of development. Embryos were harvested 48 to 72 hours after and processed for *in situ* hybridization or quantitative real-time PCR.

### *In situ* hybridization and immunostaining to tissue sections

For hybridization and immunostaining on sections, embryos were fixed in a 4% paraformaldehyde solution in PBS supplemented with 4% sucrose and 0.1 mM CaCl2, rinsed in PBS, embedded in a 15% sucrose solution, frozen in chilled isopentane before cryostat sectioning at 10–20 μm. Sections were collected on Superfrost/Plus slides (CML, France) and processed for immunolabelling or *in situ* hybridization as described previously^45^. For grafted embryos, grafted and control limbs from the same experimental embryo were positioned in the same orientation for transverse sectioning to allow comparison. For *in situ* hybridization, the following digoxigenin-labeled mRNA probes were used: chick *OSR1* and *OSR2* ^21^, chick *CXCL12* ^45^, chick *SCX* ^89^, chick *COL1a1* and *COL3a1* (produced from EST clones from ARK Genomics), mouse *Bmp4* ^54^. Chick *CXCL14* probe was produced from an RT-PCR-derived cDNA library made from E5 chick limb, using primers previously described^31^. For immunostaining, the following primary antibodies were used: mouse monoclonal anti-MF20 (Developmental Studies Hybridoma Bank, non-diluted supernatant), rabbit polyclonal anti-PDGFRα (Santa Cruz, sc-338, 1/500 dilution), rabbit polyclonal anti-TCF4 (2569, Cell signalling, 1/100 dilution). DAPI (Sigma) was diluted at 1/1000. Slides were observed with a Nikon microscope and images collected with the QCapture Pro software (QImaging) and processed using Adobe Photoshop software.

### RNA isolation, reverse transcription and quantitative real-time PCR

Total RNAs were extracted from chick limbs. 500ng to 1 µg RNAs were reverse-transcribed using the High Capacity Retrotranscription kit (Applied Biosystems). RT-qPCR was performed using SYBR Green PCR Master Mix (Applied Biosystems). Primer sequences used for RT-qPCR are listed in Table S1. The relative mRNA levels were calculated using the 2^−ΔΔCt^ method^90^. The ΔCts were obtained from Ct normalized with chick *GAPDH* or *S17* levels in each sample. For *in vitro* experiments, six cultures were used as independent RNA samples. For *in vivo* experiments, four forelimbs were used as independent RNA samples. Each sample was analysed in duplicate. Results were expressed as Standard Deviation (SD). Data were analysed by paired student t-test.

## Electronic supplementary material

Supplemental information
