# Supplementary material for: The chemokines CXCL12 and CXCL14 differentially regulate connective tissue markers during limb development
Source: Sci Rep. 2017 Dec 8;7:17279. doi: 10.1038/s41598-017-17490-z (PMC5722906; doi:10.1038/s41598-017-17490-z)
Supplement: Supplementary file 1 — Supplemental information [file 41598_2017_17490_MOESM1_ESM.pdf]

The chemokines CXCL12 and CXCL14 differentially regulate connective tissue markers during limb development

Sonya Nassari [1], Cédrine Blavet [1], Marie-Ange Bonnin [1], Sigmar Stricker [2], Delphine Duprez [1, +] and Claire Fournier-Thibault [1, +,*]

SUPPLEMENTAL INFORMATION

Table S1: list of primers

Chick qPCR primers	Forward	Reverse
CXCL12	CAAAATCCTTTCCACTCCCAACT	CCATTTGTCTCTTGCCTTACTTGTT
CXCL14	ACCCATTTTGTGTGGAGGAGA	TGCTCACCTCTCACTTTCGT
OSR1	GTGCTGAATCTCCGACTTCTATGA	GTGTAAAATCTGAAGGGCAGGAA
OSR2	CTTGCCTTGCAGGTACATCCA	TCCTGGCACTTGAAGGGTTT
COL1A2	GCAGTAACTTCATACCTAGCAACAAGC	TGCAGATGCCTCACTCACATG
COL3A1	GCGTCCTGTTGTGCCAAAA	GTTCATTCTTGCCGTGTTTCAA
COL6A1	GCATGCCTAAACAAGCGATGT	GGAACAACCCAAACCCAGATC
PDGFR α	GCTAGTGCTTGGTCGAATCC	TGTCCCTTCCACCACTTTTC
SCX	CACCAACAGCGTCAACACC	CGTCTCGATCTTGGACAGC
ID1	CCGGAGGGTCTCTAAAGTGG	GCAGGTCCCAGATGTAGTCG
ID2	GAAGAACGGCCTTTCGGAG	TCATGTTGTACAGCAGGCTCA
ID3	GCTGGAGGAACCCATGAATCT	TCCCGCAATTTGGAGTAGCA

Supplemental Figure 1: Expression of chemokine receptors during development of the chick forelimb. In situ hybridizations for *CXCR7* (A, D) and immunohistochemical detections for CXCR4 (B, F), MEP21 (C) and MF20 (E) on serial transverse sections from E5 (A-C) and E10 (D-F) chick forelimbs. *CXCR7* is expressed in CT surrounding cartilage and muscles, while CXCR4 is expressed in a sub-population of endothelial cells, as already

described (Escot et al., 2013, 2016). D: dorsal, V: ventral, a: anterior, p: posterior, r: radius, u: ulna. Bars: 200 µm in A-C, 100 µm in D-F.

Supplemental Figure 2: Expression of a dominant-negative form of *CXCR7* in vivo does not modify the expression of CT markers in the chick embryonic forelimb. (n=6). In situ hybridizations for *OSR1* (A, B), *OSR2* (D, E), *COL3A1* (G, H), *SCX* (J, K) and *CXCR7* (C, F, I, L) on serial transverse sections of control (A, D, G, J) and grafted (B, C, E, F, H, I, K, L) E10 chick forelimbs. D: dorsal, V: ventral, a: anterior, p: posterior, r: radius, u: ulna. Bar: 200µm.

Supplemental Figure 3: FGF signalling regulates the expression of CT markers independently of chemokines in the chick embryonic forelimb.
A: Experimental scheme of the grafting procedure of heparine beads coated with mouse FGF4 recombinant protein in the chick embryonic forelimb. B: 48h after the graft (n=6). FGF signalling activates *SCX* expression and represses *OSR1* and *OSR2* expression without affecting *CXCL12* and *CXCL14* expression in the chick embryonic forelimb. In situ hybridizations for *SCX* (a, b, g, h), *CXCL12* (c, d), *CXCL14* (e, f), *OSR1* (i, j) and *OSR2* (k, l) on serial transverse sections of control (a, c, e, g, j, k) and grafted (b, d, f, h, j, l) E6.5 chick forelimbs. Asterisks indicate the grafted bead. D: dorsal, V: ventral, a: anterior, p: posterior, h: humerus, r: radius, u: ulna. Bars: 250 µm in C (a-l).

Supplemental Figure 4: CXCL12 and CXCL14 regulates the expression of CT genes during chick limb development.
A: Schematic representation of the gene regulatory network contributing to dense CT differentiation in chick embryonic limb. B: A model for CXCL12 regulation of CT markers in chick embryonic limb.

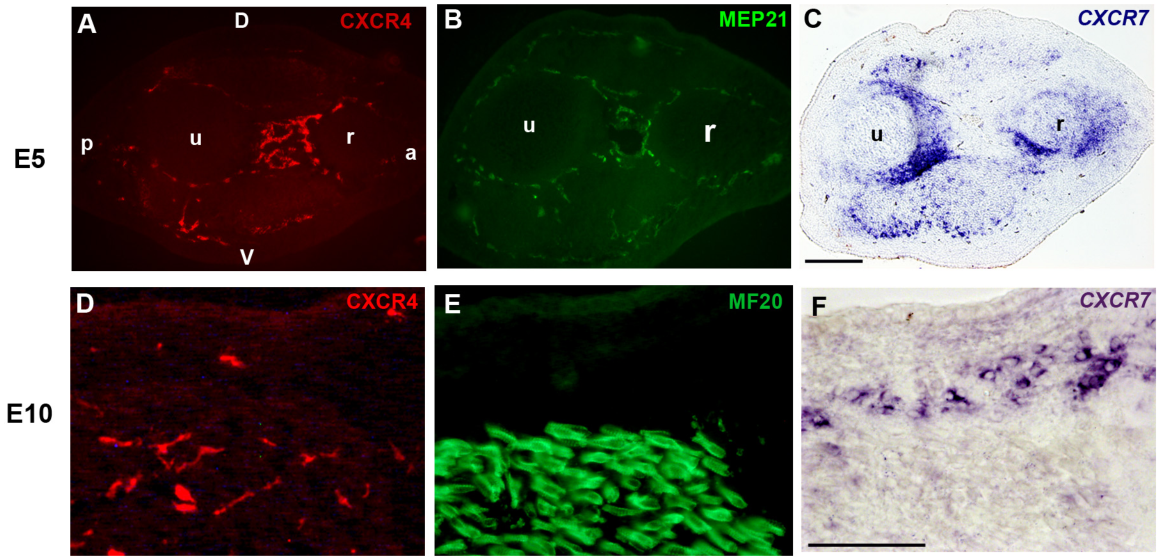

Supplemental Figure 1

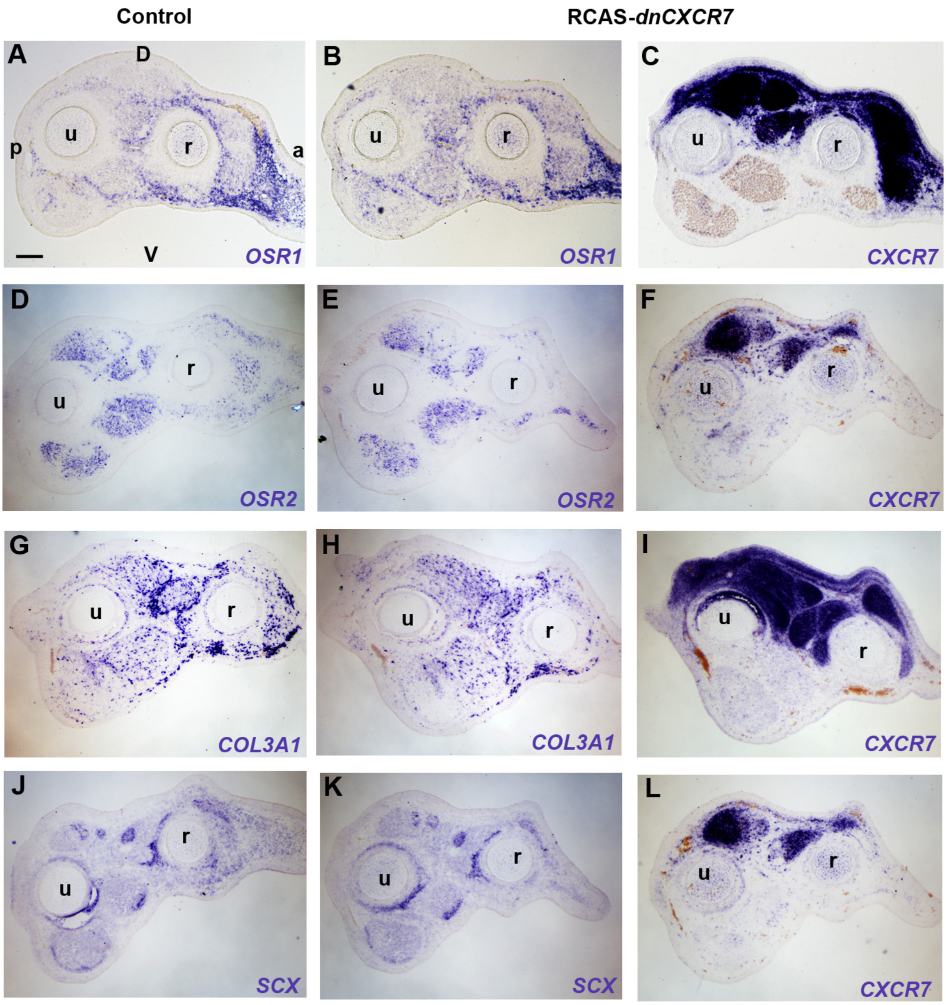

Supplemental Figure 2

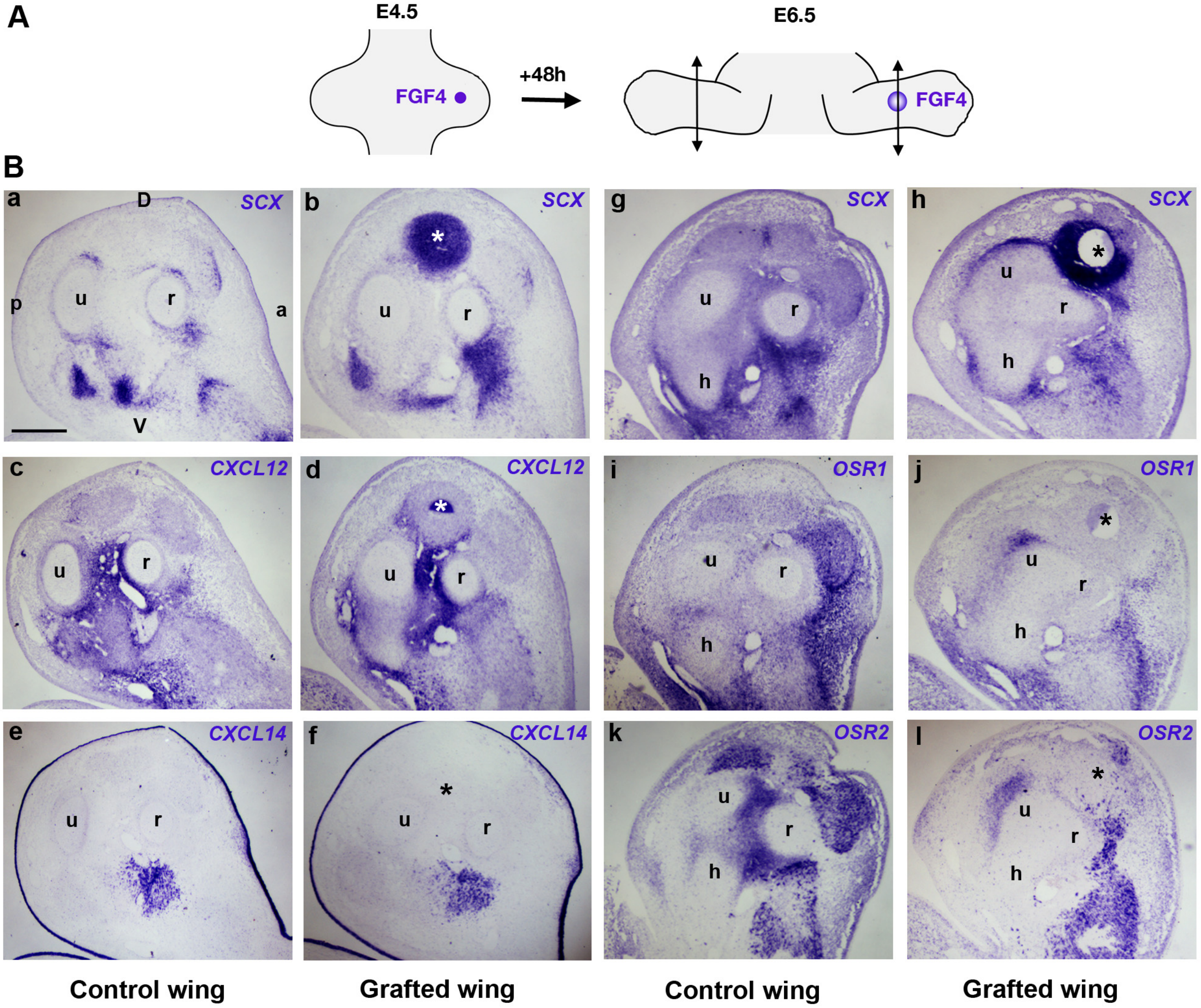

Supplemental Figure 3

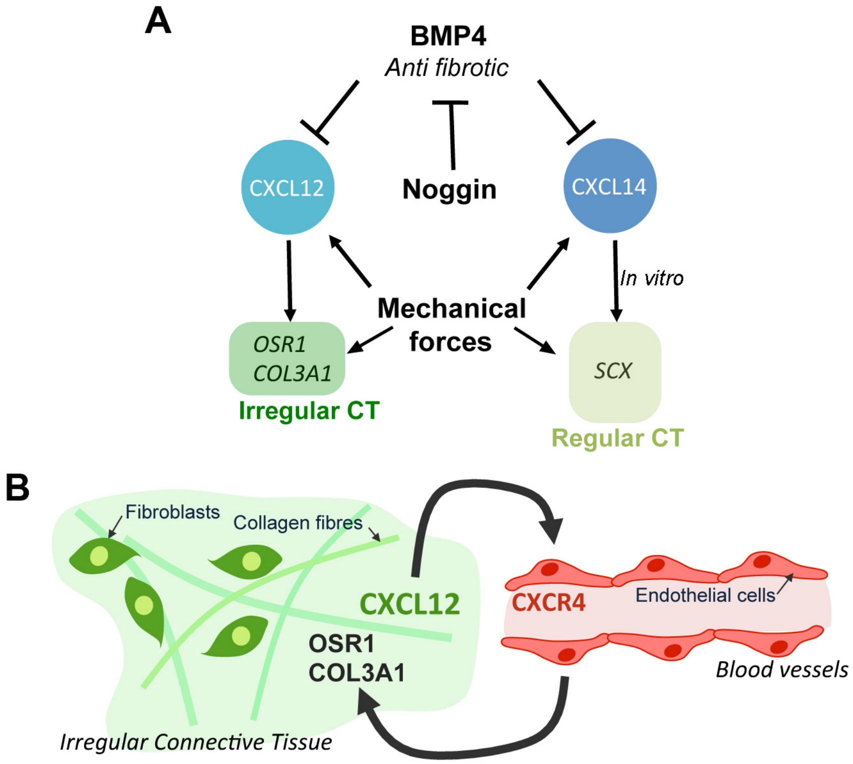

Supplemental Figure 4
